# Supplementary material for: Reaching to inhibit a prepotent response: A wearable 3-axis accelerometer kinematic analysis
Source: PLoS One. 2021 Jul 15;16(7):e0254514. doi: 10.1371/journal.pone.0254514 (PMC8281986; doi:10.1371/journal.pone.0254514)
Supplement: Attachment — Submitted filename: Response to Reviewers.pdf [file pone.0254514.s005.pdf]

PONE-D-20-39385R1
Reaching to inhibit a prepotent response: A wearable 3-axis accelerometer kinematic analysis
PLOS ONE

25 th June 2021

Response to Reviewers

A: (Answer)

Journal Requirements:

Please review your reference list to ensure that it is complete and correct. If you have cited papers that have been retracted, please include the rationale for doing so in the manuscript text, or remove these references and replace them with relevant current references. Any changes to the reference list should be mentioned in the rebuttal letter that accompanies your revised manuscript. If you need to cite a retracted article, indicate the article's retracted status in the References list and also include a citation and full reference for the retraction notice.

A: We would like to thank the journal for this note. We then reviewed all the reference list. In particular, we found in S1 Appendix bibliography a citation to a paper found at the moment of submission which is now no longer available (old citation number 6 in S1 Appendix: "Thorncroft, G. How precise is earth's gravity? https://web.calpoly.edu/~gthorncr/ME302/documents/AccuracyofGravity.pdf."). This was a paper that referred to the approximation of gravitational constant g as g=9.80665m/s^2. Considering this fact and analysing the literature we then decide to remove this reference, since g=9.80665m/s^2 is a standard approximation for gravitational constant and a reference is not necessary. In addition, we found some missing information, such as page number, in a citation of the main bibliography (old citation number 7: "Jeannerod, M. and Biguer, B. (1982). Visuomotor mechanisms in reaching within extrapersonal space."). As it is a chapter of a book which seems to be no longer distributed, and the reference does not have a central role for our work, we decided to remove it in order not to unnecessarily burdening the introduction.

Moreover, we also proofread the entire manuscript and corrected or reorganised a couple of sentences in the Abstract and Discussion sections in order to make the writing more fluid without obviously changing its content and meaning.

Reviewer #1: The authors have addressed all of my previous concerns. However, the correction to the abstract (noted within the response letter) did not appear in the revised version of the manuscript so I'd recommend that the authors double check that correction prior to publication.

A: Our apologise for this mistake. We have not corrected the abstract as previously stated.

Reviewer #2: Overall, the manuscript has been significantly improved, and most of my comments have been addressed. The authors have taken my initial feedback and made a number of important changes to their paper. I only have one remaining concern.

In my initial comments, I raised a concern over how the TPV% data was processed. The paper states that TPV% measures were excluded if they occurred outside of 5 and 95% of the trial's movement time; values

less than 5% or greater than 95% would represent technical failure rather than true human movement. I asked how the authors could be confident that values within 5 and 95% were valid measurements, and they responded with a statistics analogy to the arbitrary alpha level of .05. Yes, .05 is indeed arbitrary, but my concern has nothing to do with statistics. It has to do with how the data was processed, and data processing should not be arbitrary. I will try and clarify my earlier comment here.

My concern centers around the authors' use of "technical failure." To me, technical failure would mean that something went wrong with either the equipment, the software, data processing, or a combination of all three, which ultimately led to an incorrect, or erroneous, TPV% value. The current solution was to simply omit values less than 5% or greater than 95%, but as the authors themselves state, these are arbitrary numbers. My concern is, how do you know that values within 5-95% weren't also erroneous? For example: in a single trial, a person might have reached their TPV% at 50% of the total movement time, but the value that was actually calculated came out to be 87% (due to a technical failure). This value of 87% is an error, but since it falls within the 5-95% boundaries, it will be included in the data set. How do the authors know that this wasn't happening?

If I were to draw a simple analogy, electromyography (EMG) collections typically use a band pass filter (example: 10-500 Hz; anything below 10 Hz and anything above 500 Hz will be omitted from the signal). This serves to remove any low frequency noise, such as movement, and high frequency noise from the signal. However, electrical noise with a frequency range within 10-500 Hz can still contaminate the signal. How do the authors know that these "technical failures" (i.e. noise) weren't present in the 5-95% acceptable range?

A: We would like to thank the reviewer for his/her careful considerations, and for clarifying this doubt about our choice to exclude TPVs outside the 5-95% range. We apologize for not fully understanding this question, which we will now attempt to answer more adequately. There seems perhaps to be some misunderstanding about the difference between data preprocessing and analysis.

Taking up the analogy with the EMG data, we can say that the bandpass filters that remove noise from the signal correspond to what we did in the preprocessing phase of the raw acceleration values collected by the accelerometer, as reported in detail in S1 Appendix. This preprocessing phase was meant to remove potential noise.

From the resulting preprocessed data, we computed the TPV index, which represents participants' performance in each trial. Our apologizes for any misunderstanding caused by our improper use of "technical failures", which primarily referred to "*numerical errors, in cases where the detrend function was not sufficient to remove their effect on the signal",* whereby "numerical errors" refer to those resulted from the application of an integration function due to a possible bias (i.e., additive noise) present in the acceleration (as written in Section "Materials and methods – Kinematics measures"). Moreover, as in all cases of human performance measurement, the TPV is variable and potentially resulting from human errors (e.g., in the cases where participants made extra-task movements and did not precisely keep following the instructions). More generally, we decided to remove the whole observations (not part of the acceleration signals) with a TVP outside the 5-95% range. Indeed, based on the existing literature, we can expect TPVs around the average value of 50% of movement duration. Therefore, those smaller than 5% and greater than 95% seem unlikely related to the task-related human reaching movements that are required by our specific task and paradigm (an adapted Go/No-Go paradigm).

In order to clarify this aspect, we rephrased this part of the manuscript as it follows:

Ultimately, we aimed to exclude possible extreme TPV% values that would be due to numerical errors, in cases where the detrend function was not sufficient to remove their effect on the signal. Moreover, we aimed to remove those observations with TPV% values that were unlikely related to task-related human reaching movements, but rather potentially ascribable to extra-task movements. For these reasons, the a-priori inclusion criteria for valid TPV% values comprehended those between 5% and 95%. At the end of this procedure, we excluded 59 out of 2,962 trials.

As far as we correctly understand your comment, we are not able to check whether additional extra-task movements occurred and influenced the accepted TPVs, which is true for many psychology experiments, whereby average data are meant to flatten the effect of potential human-related noise. However, your suggestions made us wonder whether, in future studies, we might address this issue by including video recordings and offline coding of the experimental sessions, to further check for visible cases of extra-task movements. We are used to do so when testing infants and children, whose performance is particularly variable. We did not employ video recordings with the present study, as neurotypical adults are usually quite good at following experimental instructions and procedures. We now acknowledge this interesting point in the Discussion:

Moreover, future studies might include video recordings and offline coding of the experimental sessions, thus checking for potential cases where participants show extra-task movements that could result in anomalous trials.

Notably, the exclusion of extreme TPVs made us remove only 59 out of 2.962 trials (less than the 2% of observations). To further explore the effect of removing TPVs out of the 5-95 range, please see below the variable distributions (a) before and (b) after (equal to the one reported in Figure 3 of the manuscript) removing the TPVs outside the 5-95% range. Notably, the removal of extreme TPVs did not affect the distribution shape nor the difference between values observed in the conditions of interest (Dominant vs Non-dominant). Indeed, when we run the model comparison analysis (c) before and (d) after removing the TPVs outside the 5-95% range, the Condition effect appears robust. In conclusion, we are confident that the present manuscript presents robust and valuable results, and the actual limitations are properly described to inform future steps.

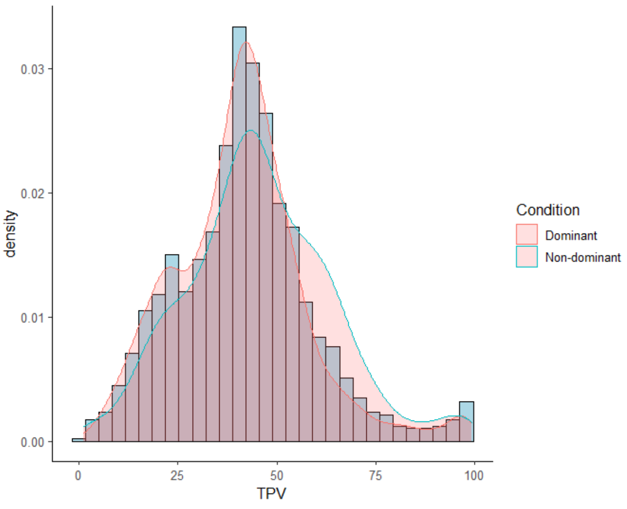

Figure (a): all TPVs

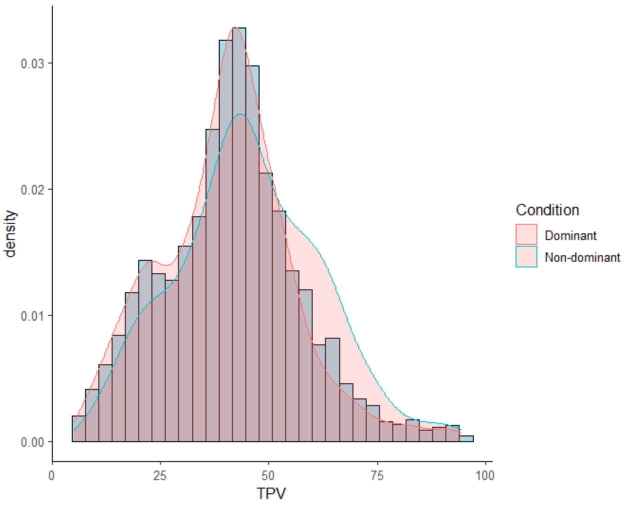

Figure (b): TPVs in 5-95% range

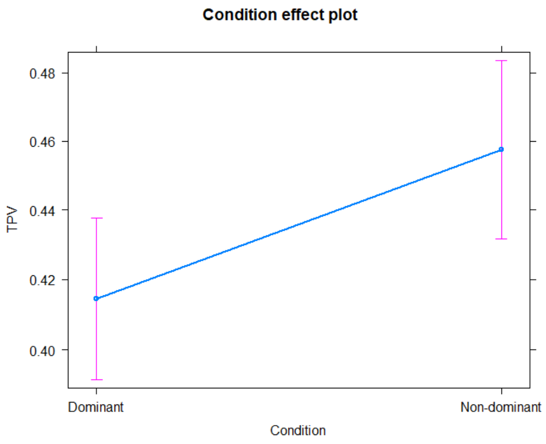

Figure (c): all TPVs

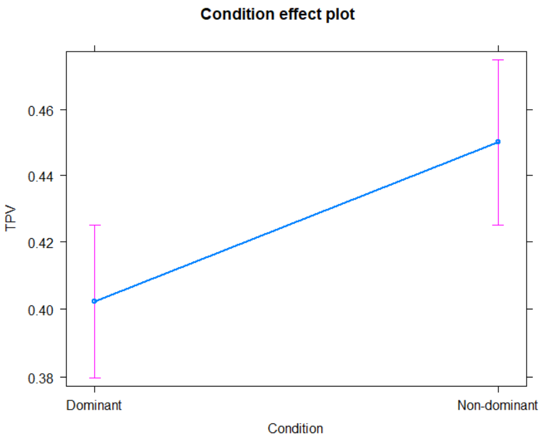

Figure (d): TPVs in 5-95% range
